# Remarks on the Institutes of Dental Science

**Published:** 1865-12

**Authors:** C. W. Spalding


### REMARKS,

**Introductory to the course on the Institutes of Dental Science, in the Ohio College of Dental Surgery.**

BY C. W. SPALDING, D. D. S.

Gentlemen: —I appear before you for the purpose of giving you a course of instruction in those branches of Dental education, comprehended in the term "*Institutes of Dental Science.*"

By the term Institutes of Dentistry, we understand the elucidation of those principles of general medicine and therapeutics which lie at the foundation of all true pathological science, and in the application of these general principles to the treatment of special dental diseases.

From this chair, it is expected, will be taught those things pertaining to an enlightened dental practice which are not specially treated of by my colleagues in this institution, and will include especially, etiology, pathology, diagnosis and prognosis, to be followed by such a system of therapeutics as I have myself acquired from books and from intercourse with my fellow practitioners; verified by my own experience and enlarged and extended by my own reasonings and by observation.

My course will necessarily embrace, in Dental Pathology proper, the subjects of salivation, necrosis, exostosis, neuralgia, both sympathetic and that arising in the dental organs themselves; odontalgia in its various forms, including the treatment of exposed pulps, both for their preservation and destruction; alveolar abscess from whatever cause it may arise, and in short all those diseases, from their incipiency to their consummation, to which the dental organs are known to be liable.

More or less of physiology and histology, both general and special will be unavoidably treated of, including first and second dentition; also, anæsthesia, dental materia medica, both Allopathic and homœpathic, dental anomalies and cleft palate.

Many other points not here enumerated will be touched upon in my endeavor to impart to you such information as will be useful to you in your daily practice. While I shall speak mainly of Dental science, I shall at the same time introduce to your notice more or less of the art or arts of dentistry.

Science and Art, especially in the practice of your chosen profession, are, or should be inseparable. They should go hand in hand, and be required to keep an even pace with each other, lest on the one hand mere theory becomes predominant unaccompanied by that degree of executive ability and skill necessary to a proper ultimation of the conceptions of the mind, or lest on the other hand, the education of the hand and the development of the manipulative faculties shall so far outstrip the cultivation of the mind, as shall cause the practitioner of dentistry to become an opera*tive* rather than an opera*tor*, and the occupation of the dentist shall sink below the dignity of a learned profession.

Previous to the establishment of Dental Colleges, the arts of dentistry were cultivated almost to the exclusion of the sciences, and even now, one can hardly look abroad in any large community without discovering some remains of the old custom of introducing men into the profession as soon as they have acquired any considerable ability to handle an instrument or extract a tooth.

Do not understand me, however, as underrating the importance of operative skill. It is not only important, but it is absolutely necessary to the successful practice of our useful and difficult art.

We may cram the brain with theories until the man sparkles all over with science, but he will find them of little value unless they are at the same time joined to that education of the hand that will alone enable him to ultimate them in practice.

Therefore, what you should seek to acquire in your course in this institution, is that operative and manipulative skill, which shall be guided by an intelligent understanding and appreciation of all those basal principles which are designated by the term "*Institutes of Dental Science.*"

